# Measuring the Affordability of Nutritious Diets in Africa: Price Indexes for Diet Diversity and the Cost of Nutrient Adequacy

**DOI:** 10.1093/ajae/aay059

**Published:** 2018-08-13

**Authors:** William A. Masters, Yan Bai, Anna Herforth, Daniel B. Sarpong, Fulgence Mishili, Joyce Kinabo, Jennifer C. Coates

**Keywords:** Food prices, diet quality, diet diversity, nutrient adequacy, CPI

## Abstract

Policies and programs often aim to improve the affordability of nutritious diets, but existing food price indexes are based on observed quantities that may not meet nutritional goals. To measure changes in the cost of reaching international standards of diet quality, we introduce a new cost of diet diversity index based on the lowest-cost way to include at least five different food groups as defined by the widely used minimum dietary diversity for women (MDD-W) indicator and compare that to a Cost of Nutrient Adequacy indicator for the lowest-cost way to meet estimated average requirements of essential nutrients and dietary energy. We demonstrate application of both indexes using national average monthly prices from two very different sources: an agricultural market information system in Ghana (2009–14) and the data used for national consumer price indexes in Tanzania (2011–15). We find that the cost of diet diversity index for Ghana fluctuated seasonally and since mid-2010 rose about 10% per year faster than national inflation, due to rising relative prices for fruit, which also drove up the cost of nutrient adequacy. In Tanzania there were much smaller changes in total daily costs, but more adjustment in the mix of food groups used for the least-cost diet. These methods can show where and when nutritious diets are increasingly (un)affordable, and which nutritional criteria account for the change. These results are based on monthly national average prices, but the method is generalizable to other contexts for monitoring, evaluation, and assessment of changing food environments.

*JEL codes:* I15, Q11, Q18.

Price indexes for traded food commodities are widely reported by international agencies such as the Food and Agricultural Organization (FAO 2018), while local wholesale and retail prices are collected and used in almost all countries to monitor producer prices, market conditions, overall inflation and living standards (World Bank 2017a, 2017b). Formulas to aggregate individual items into price indexes were first introduced more than 300 years ago (Diewert 1993), with continued changes needed to reflect what and how goods and services are consumed (Diewert, Greelees, and Hulten 2010; Rippy 2014).

The purpose of most price indexes is to capture changes in the cost of what is actually bought and sold, which can vary greatly in nutritional quality over time and across groups (Beatty, Lin, and Smith 2014; Clements and Si 2018). To make nutritious diets more affordable, policies and programs may aim to lower the relative cost of more nutritious foods, and sometimes also raise the cost of less healthy items. The aim of this paper is to develop improved indexes for the cost of a nutritious diet relative to other prices in the African context, where healthier foods such as dairy, eggs, fruits and vegetables vary greatly in price (Green et al. 2013; Harttgen, Klasen, and Rischke 2016).

The oldest and most widely used approach to measuring the cost of healthy diets is the cost of nutrient adequacy. Soon after the discovery of essential nutrients, Stigler (1945) pioneered the development of linear programming methods for calculating how much of each food would be needed to meet recommended intake of each required nutrient at lowest total cost. Allen (2017) uses this kind of price index for poverty measurement, and others use these least-cost diets to track the cost of nutrients over time (O’Brien-Place and Tomek 1983; Håkansson 2015; Omiat and Shively 2017), make comparisons across countries (Chastre et al. 2007) or compare actual choices to least-cost diets within a country (Jensen and Miller 2010; Maillot et al. 2017). Least-cost diets are often used to make nutritional recommendations for low-income consumers. At the United States Department of Agriculture (USDA), the “Minimum-Cost Food Plan” proposed for people facing extreme poverty during the depression of the 1930s (Cofer et al. 1962) evolved with the use of linear programming into the Thrifty Food Plan (TFP) to calculate and justify the amount of money provided in food stamps and supplemental nutrition assistance for low-income Americans (USDA 2017). The same method is used internationally, for example to make recommendations in Denmark (Parlesak et al. 2016) and the Netherlands (Gerdessen and De Vries 2015). One of the most important uses for least-cost diets is to help nutrition assistance programs meet specific needs of children and other vulnerable groups, as in the *Cost-of-the-Diet* approach developed by Save the Children UK and others (Chastre et al. 2007; Deptford et al. 2017; Akhter et al. 2018), and *Optifood* developed by the London School of Hygiene and Tropical Medicine and others (Optifood 2012; Vossenaar et al. 2017).

Our aim in this paper is to extend the literature on the cost of nutritious diets to diversity among food groups. Consuming foods from a variety of different categories is often seen as desirable for reasons beyond nutrient adequacy, leading nutritionists to standardize diet diversity measurement by grouping foods in terms of various functional characteristics. The specific criteria we use in this paper are from the Minimum Diet Diversity for Women (MDD-W) indicator for women of reproductive age (FAO and FHI360 2016; Martin-Prevel et al. 2017). MDD-W is defined as consuming foods from at least five out of ten specific food groups during the previous day or night. This has been linked to nutrient adequacy in several low-income countries (Arimond et al. 2010) and may confer additional health benefits associated with phytochemicals and other diet qualities in addition to nutrients (Shiraseb et al. 2016). Different functional groups and thresholds could be used for other populations at risk of malnutrition. For example, the standard international indicator for dietary diversity in children aged 6–23 months is whether they consumed at least one item from at least four out of seven specific food groups in the previous day or night (World Health Organization [WHO] and UNICEF 2007; UNICEF 2016). Dietary diversity based on the number of food groups consumed in the past 24 hours is operationally useful for policy analysis and program management, since it can be measured quickly using a list-based method, whereas the volume of food consumed and its nutrient composition are much more difficult to quantify and analyze. Designing a food price index around this criterion allows us to determine whether including diverse foods in the diet is increasingly (un)affordable for consumers at each time and place, to reach a minimum number of groups or to include at least one item from every food group.

## Methods

To track changes in the cost of nutritious diets with broad relevance for the adult population, we compute a price index defined around the MDD-W and compare that to the corresponding cost of nutrient adequacy, using monthly national average food prices in Ghana and Tanzania. We refer to the two measures as the cost of dietary diversity (CoDD), defined as the least-cost foods needed to meet the MDD-W, and the cost of nutrient adequacy (CoNA), defined as the least-cost foods needed to meet average nutrient requirements. Both are computed relative to all other prices in the local economy and converted to constant US dollars at purchasing-power parity (PPP) exchange rates. This provides comparable inflation-adjusted price indexes, measuring the cost of reaching these two nutritional standards relative to all prices in the economy. Some foods appear in our nutritional price index also appear in the PPP price level but with different weights. Overall inflation is based on average expenditure in each country as computed by the International Comparisons Project (World Bank 2017a), while our indexes are based on each food’s contribution to meeting international nutrition standards.

Our price index for the cost of dietary diversity, CoDD, is defined as the least expensive way of acquiring some food from each food group needed to reach the MDD-W. To aggregate over groups, we provide two distinct measures: a simple CoDD1 counts only the least-cost food in the fifth least expensive food group, while a broader CoDD2 counts the average of the least-cost food in all food groups. CoDD1 reflects a narrow version of the MDD-W defined so that dietary diversity can be achieved just by reaching the fifth group, while CoDD2 reflects a broader version in which consumers include all food groups with equal frequency. The CoDD concept is based on rank order optimization based on food prices within groups, defined as:

(1) $$\begin{array}{l} \text{CoDD}1=\text{min}5\{\text{min}\{p_{i1}\},\\ \text{ min}\{p_{i2}\},\ldots ,\text{min}\{p_{im}\}\} \end{array}$$

(2) $$\begin{array}{l} \text{CoDD}2=\text{ave}\{\text{min}\{p_{i1}\},\\ \text{ min}\{p_{i2}\},\ldots ,\text{min}\{p_{im}\}\} \end{array}$$

where min5 denotes the fifth lowest of all *m* food groups, and *p_ij_* is the price of item *i* in the *jth* food group. By definition, the MDD-W indicator and hence CoDD price indexes make no reference to the quantities consumed above one serving of each food, so prices and hence the overall index are standardized to cost per kcal. Additional information in the supplementary online material is provided on costs per gram. Also by definition only the least-cost food within each group is included, so the foods included in CoDD are not necessarily a positive description of what people actually consume or a normative prescription for what they should consume. Instead, CoDD1 provides a lower bound on the cost of including the fifth group to just meet the MDD-W threshold, while CoDD2 provides a lower bound on the cost of acquiring some of each MDD-W food group, thereby tracking changes in access to foods needed to meet that nutritional standard.

As a benchmark for comparison we use the same data to compute the cost of nutrient adequacy (CoNA), defined as the minimum cost of foods that meet all known requirements for essential nutrients and dietary energy requirements for an adult woman of reproductive age. While we focus on women of reproductive age for both CoDD and CoNA, CoNA can be computed for other age or population groups, such as young children, simply by using energy and nutrient requirements specific to those groups. CoNA can be written formally as:

(3) $$CoNA:\text{minimize }C=\sum_{i}p_{i}\times q_{i}$$

Subject to:

(4) $$\sum_{i}a_{ij}\times q_{i}\geq EAR(\text{j}=1,2,3,\ldots ,\text{n})$$

(5) $$\sum_{i}q_{ie}\times q_{i}=E$$

(6) $$q_{1}\geq 0,q_{2}\geq 0,\ldots ,q_{i}\geq 0$$

Here the quantity of the *jth* nutrient in food *i* is denoted *a*_ij_, which multiplied by its quantity consumed (*q*_i_) must meet the population’s estimated average requirement (*EAR*) for nutrient *j*, at lowest total cost given all prices (*p*_i_) within the further constraint of overall energy balance (*E*) which for convenience we set at 2,000 kcal/day. There are twenty-one known essential nutrients, but for nutritional adequacy we drop vitamin D and cholesterols, which can be synthesized in human bodies, and iodine and molybdenum due to lack of data in the food composition databases, leaving *n* = 17 nutrient constraints plus a constraint for energy balance. This computation provides a lower bound on the cost of meeting the EARs, allowing us to track changes in the cost of limiting nutrients much as the CoDD tracks changes in the cost of limiting foods.

For both CoDD and CoNA we report which foods would be needed to meet each nutritional target at lowest cost, thereby tracking changes in access to that international standard. By defining “access” to mean a lower bound on total cost, these price indexes deliberately differ from what any group might *actually* consume (for which we would use a consumption price index), or *should* consume (in the sense of a recommended diet). Actual diets may exceed or fall short of any given nutritional standard, and methods designed to make dietary recommendations include additional stipulations to obtain a locally acceptable, “normal” diet (Chastre et al. 2007; Deptford et al. 2017; Cost of Nutritious Diets Consortium 2018). For our purposes, we avoid specifying local eating habits and cultural norms so as to compare food prices only with respect to international standards for nutritional content. The foods whose prices are included in CoDD and CoNA are the lowest cost way to meet those standards at each location, which may point to foods that are currently consumed in small quantities but could play a larger role in local diets if culinary practices were to change in response to relative prices. Apart from those two price indices presented here, parallel work is under way to construct nutritionally weighted consumer price indexes (nCPI) that would reflect nonmarket (dis)utilities from the foods actually consumed, and to construct globally relevant cost of a recommended diet (CoRD) indexes that would reflect normative dietary guidelines published by national or international agencies (Herforth 2017).

The focus of CoNA is the cost of nutrients, which is reflected in their shadow prices (*SP*) defined as the cost increase associated with increasing each constraint by one unit:

(7) $$SP_{j}=\frac{\partial C*}{\partial EAR_{j}^{+}}$$

where C* denotes the (minimum) cost of the CoNA diet. *SP_j_* is the *SP* of nutrient *j* (or daily dietary energy), and $EAR_{j}^{+}$ refers to one unit increase in EAR of nutrient *j* (or daily dietary energy). Since units of measure for nutrients may differ, we construct a semielasticity denoted *SP’* as increment in cost of the CoNA diet when the constraint is increased by 1%, expressed as:

(8) $$SP_{j}^{\prime }=\frac{\partial C*}{\%\Delta EAR_{j}^{+}}$$

The sum of ${\text{SP}}_{j}^{\prime }(\sum_{j}SP_{j}^{\prime }\text{ or }SP’)$ of all seventeen nutrients and dietary energy equals to the change of CoNA when all nutritional and energy constraints are increased by 1% together. For ease of comparison with CoNA itself we report *SP’* multiplied by 100, which we refer to as the shadow price contribution (SPC) of nutrient *j* or dietary energy:

(9) $$SPC_{j}=SP_{j}^{\prime }\times 100$$

Similarly, we further calculated the shadow price elasticity (SPE) of nutrient *j* defined as the percentage change of the cost of the CoNA diet package evaluated at the optimal basis in response of 1% increase in EAR of nutrient *j*:

(10) $$SPE_{j}=\frac{\%\Delta C*}{\%\Delta EAR_{j}^{+}}$$

The SPE is useful to identify the limiting nutrients for which the level of EAR contributes the most to CoNA at each time and place. It measures the change in total cost associated with a marginal change in each nutrient requirement, thereby revealing the degree to which that particular requirement accounts for differences in the cost of acquiring all essential nutrients.

Calculations for all equations were completed in R and resulting index values exported to Stata or Excel for visualization purposes, with model code and data for replication posted online at the project website referenced in this paper’s acknowledgements.

## Data

Our empirical application draws on four main data sources. Food price data are national average monthly food prices in Ghana between March 2009 and December 2014, and in Tanzania between January 2011 and December 2015. These were collected by national authorities and cover a total of 34 distinct foods in Ghana and 71 in Tanzania. Prices for each item are unweighted averages over a variety of retail markets, covering all ten regions of Ghana and all twenty-one regions of mainland Tanzania. Primary data collection was conducted by the Ministry of Food and Agriculture (MoFA) in Ghana for their market information system, and by the National Bureau of Statistics (NBS) in Tanzania for the purpose of inflation monitoring. In this paper we deliberately use data with different institutional origins to show the range of applicability for these indexes, recognizing that differences between countries also reflect differences in data-collection methods. There were no missing values in the Tanzania data, but for Ghana there are missing observations for soybeans (Feb 2010) and mango (Aug, Sep, and Oct 2009; Feb 2011; Sep and Oct 2013). To complete the data set for results shown here we impute prices by carry-over from the previous month. This method is unlikely to truncate seasonal extremes, as mangoes in Ghana generally mature between May and August, with some varieties in southern Ghana also maturing between December and February (MoFA 2017).

To compute the price indexes, the price of each food was converted from reported units, such as price per dozen eggs, to cost per unit of weight and/or of dietary energy of the edible portion, and then converted to a common currency and adjusted for inflation by purchasing-power-parity (PPP) conversion factor provided by the World Bank (2017a). We excluded most processed foods and classified foods into one of ten mutually exclusive food groups based on the FAO and FHI360 (2016) guidelines for calculating MDD-W: (1) Grains, white roots and tubers, and plantains, (2) pulses, (3) nuts and seeds, (4) dairy, (5) meat, poultry and fish, (6) eggs, (7) dark green leafy vegetables, (8) vitamin A-rich fruits and vegetables, (9) other vegetables, and (10) other fruits. Additional foods that people might consume are not included in the MDD-W calculation, notably oils and fats, sweets and other foods, beverages other than dairy, condiments and seasonings. The available price data for Ghana cover twenty-six foods from eight of the ten MDD-W food groups, and price data for Tanzania cover forty-six foods from all ten groups. The missing food groups in Ghana are dairy and dark green leafy vegetables. We use these data to highlight that data gaps are often present in food price data monitoring systems, where staple crops are the focus and nutritionally important foods may be missing. The MDD-W offers a quick way to assess whether monitoring systems cover sufficient diversity to estimate the cost of nutritious diets: if there are no or few items representing each of the ten MDD-W categories, then those data gaps should be corrected. Based on these analyses, we collaborated with the Ghana MoFA to add nutritious food items to their food price monitoring system (Nortey 2017). By definition, cooking oil is not included in the MDD-W or CoDD, but we do include it as a source of dietary energy for CoNA.

Additional data required for the calculation of CoNA include the nutrient composition and edible portions of each food as purchased, obtained from the two standard sources: FAO’s West African Food Composition Table (Stadlmayr 2012), complemented by the U.S. National Nutrient Database for Standard Reference (USDA 2013). Detailed food lists with nutrients compositions for both countries are presented in online supplementary material tables A4 and A5. Nutrient requirements are obtained from the estimated average requirements (EARs) for adult women from nineteen to thirty years old, as specified in dietary reference intakes (DRIs) developed by the U.S. Institute of Medicine of the National Academies. EAR, defined as the average daily nutrient intake level estimated to meet requirements at least half of the healthy individuals in a group, is the primary reference point for assessing the adequacy of estimated nutrient intakes of groups and is a tool for planning intakes for groups (Institute of Medicine 2006). A detailed table with energy and nutrients criteria is presented in online supplementary material table A3.

## Results

Descriptive statistics for prices per unit of dietary energy are summarized in tables 1 and 2. The underlying descriptive statistics for prices per unit of weight are provided in the online supplementary material, as tables A3 and A4. For Ghana, we have a total of 70 monthly observations from March 2009 to December 2014 for twenty-five items, and fifty-six monthly observations from May 2010 to December 2014 for paddy rice. Of these, twelve food items are in the starchy staple group, reflecting the strong focus of data collection efforts on that category. The average price of each item per 1,000 kcal ranges widely, from $0.26 for maize to $20.77 for tomatoes, while prices per kg range from $0.53 for cassava to $8.90 for eggs shown in online supplementary material table A1. The volatility of food prices over time, as represented by coefficient of variation (CV), varies widely from 0.07 for eggs to 0.36 for mangoes.

**Table 1:** Descriptive Statistics for Monthly Food Prices per 1,000 kcal – Ghana (2011$)

Food Groups	No.	Foodstuffs	Obs.	Mean	Std. Dev.	CV	Min	Max
Grains, white roots and tubers, and plantains	1	Cassava	70	0.33	0.07	0.20	0.23	0.48
	2	Cocoyam	70	1.07	0.24	0.23	0.71	1.62
	3	Kokonte	70	0.38	0.06	0.17	0.27	0.54
	4	Garri	70	0.44	0.07	0.17	0.34	0.72
	5	Rice (imported)	70	0.73	0.12	0.16	0.60	1.09
	6	Rice (local)	70	0.52	0.06	0.12	0.42	0.75
	7	Maize	70	0.26	0.05	0.18	0.19	0.40
	8	Millet	70	0.39	0.05	0.13	0.31	0.51
	9	Paddy Rice	56	0.40	0.13	0.32	0.24	0.86
	10	Plantains	70	1.47	0.49	0.33	0.91	3.38
	11	Sorghum	70	0.37	0.04	0.11	0.29	0.47
	12	Yam	70	1.04	0.17	0.16	0.76	1.48
Pulses	13	Cowpea	70	0.61	0.10	0.17	0.43	0.85
	14	Soybeans	70	0.29	0.07	0.24	0.13	0.47
Nuts and seeds	15	Groundnuts (shelled)	70	0.58	0.11	0.19	0.40	0.79
Meat, poultry, and fish	16	Anchovies	70	4.83	1.04	0.22	2.43	8.92
	17	Tilapia (dried)	70	2.53	0.61	0.24	1.03	4.32
	18	Herring (smoked)	70	1.99	0.45	0.22	1.27	3.45
Eggs	19	Eggs	70	6.23	0.44	0.07	5.22	7.58
Vitamin A-rich vegetables and fruits	20	Mangoes	70	1.41	0.51	0.36	0.64	2.94
Other vegetables	21	Eggplant	70	9.16	2.37	0.26	4.78	16.55
	22	Onions (large)	70	8.95	2.90	0.32	4.20	14.51
	23	Tomatoes	70	20.77	6.88	0.33	10.09	39.91
Other fruits	24	Bananas	70	1.90	0.37	0.20	1.15	2.84
	25	Oranges	70	2.94	0.90	0.31	1.20	6.72
	26	Pineapples	70	2.94	0.32	0.11	2.29	3.87

*Note*: Authors’ calculations, from Ghana Ministry of Food and Agriculture file data. Two food groups in the MDD-W are not represented in this data set: Dairy, and Dark Green Leafy Vegetables. Data were imputed by carry-over from the previous month to fill missing observations for soybeans (Feb 2010) and mango (Aug, Sep, and Oct 2009; Feb 2011; Sep and Oct 2013). Kokonte and garri are forms of processed cassava.

**Table 2:** Descriptive Statistics for Monthly Food Prices per 1,000 kcal–Tanzania (2011$)

Food Group	No.	Foodstuff	Obs.	Mean	Std. Dev.	CV	Min	Max
Grains, white roots and tubers, and plantains	1	Cassava (dried flour)	60	0.60	0.07	0.11	0.48	0.79
	2	Cassava (fresh)	60	0.77	0.07	0.09	0.60	0.90
	3	Plantain	60	1.64	0.09	0.05	1.45	1.90
	4	Finger millet	60	0.68	0.11	0.17	0.50	0.87
	5	Maize flour	60	0.47	0.06	0.12	0.37	0.63
	6	Potatoes – round	60	2.25	0.13	0.06	1.97	2.63
	7	Rice	60	0.74	0.12	0.16	0.57	0.98
	8	Sweet potatoes	60	1.70	0.14	0.08	1.46	1.97
	9	Wheat flour	60	0.62	0.04	0.06	0.56	0.71
	10	Maize (white)	60	0.31	0.04	0.12	0.24	0.41
Pulses	11	Soybeans	60	0.65	0.03	0.04	0.59	0.70
	12	Lentils	60	1.28	0.12	0.09	1.08	1.48
	13	Beans (red)	60	0.78	0.04	0.05	0.72	0.87
Nuts and seeds	14	Groundnuts	60	0.66	0.05	0.08	0.58	0.78
Dairy	15	Milk (fresh)	60	2.89	0.16	0.05	2.38	3.07
	16	Milk (powdered)	60	7.99	0.38	0.05	7.02	8.72
Meat, poultry, and fish	17	Beef sausage	60	4.32	0.08	0.02	4.18	4.54
	18	Beef with bones	60	3.92	0.19	0.05	3.47	4.43
	19	Beef without bones	60	1.11	0.04	0.04	1.01	1.26
	20	Dried sardines	60	5.99	0.46	0.08	5.12	6.91
	21	Goat meat	60	9.51	0.38	0.04	8.37	10.19
	22	Chicken (live, industrial)	60	6.57	0.31	0.05	5.6	6.99
	23	Pork meat	60	3.17	0.28	0.09	2.45	3.63
	24	Chicken (live, traditional)	60	11.9	0.79	0.07	9.94	13.26
Eggs	25	Eggs (layers)	60	8.42	0.28	0.03	7.89	8.88
	26	Eggs (traditional)	60	11.81	0.69	0.06	10.3	12.66
Dark green leafy vegetables	27	Amaranth (mchicha)	60	5.74	0.57	0.10	4.85	6.81
Vitamin A-rich vegetables and fruits	28	Carrots	60	7.05	0.69	0.10	6.01	9.08
	29	Mangoes	60	4.46	0.63	0.14	2.97	6.06
	30	Papaya	60	5.63	0.50	0.09	4.71	6.64
Other vegetables	31	Tomatoes (bitter)	60	8.86	0.46	0.05	7.85	10.72
	32	Eggplant	60	9.44	0.49	0.05	8.47	10.83
	33	Cabbage	60	2.80	0.27	0.10	2.30	3.48
	34	Green peas	60	24.78	1.74	0.07	20.72	28.40
	35	Green bell pepper	60	16.46	0.92	0.06	14.78	19.16
	36	Okra (ladies fingers)	60	11.28	0.75	0.07	9.97	13.25
	37	Onions	60	6.43	0.77	0.12	5.21	8.86
	38	Tomatoes (red)	60	10.44	1.19	0.11	8.36	13.53
Other fruits	39	Apples (imported)	60	19.58	1.62	0.08	15.85	23.62
	40	Avocado	60	1.91	0.12	0.06	1.67	2.18
	41	Coconut (mature)	60	5.52	0.51	0.09	4.78	6.85
	42	Lemons	60	11.75	2.03	0.17	8.26	17.99
	43	Limes	60	15.62	2.87	0.18	12.00	23.57
	44	Oranges	60	4.43	0.46	0.10	3.47	5.63
	45	Pineapples	60	6.66	0.65	0.10	5.54	7.98
	46	Sweet banana	60	3.35	0.28	0.08	2.71	3.91

*Note:* Authors’ calculations, from Tanzania Bureau of Statistics file data.

For Tanzania, we have a total of sixty monthly observations over five years from January 2011 to December 2015 for forty-six items spanning ten food groups as the final data base for index calculation. Starchy staples group, as the largest food group in terms of the number of food items, contains 10 items. Average prices per 1,000 kcal range from $0.31 for white maize to $24.78 for green peas, and prices per kg range from $1.11 for white maize to $39.56 for powered milk. The volatility of prices ranges from a CV of 0.02 for beef sausage and goat meat to 0.18 for limes.

**Figure 1:**
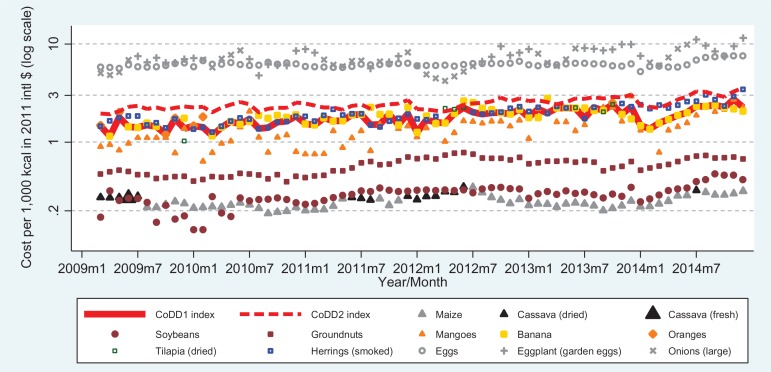
Cost of diet diversity in Ghana, March 2009–December 2014. *Note:* Foods shown are the least-cost item in their food group, as defined by the minimum dietary diversity for women (MDD-W) indicator, ranked in cost per unit of dietary energy. CoDD1 is the cost of reaching the fifth group, and CoDD2 is the cost of including all groups. Groups in ascending order of usual cost are starchy staples (maize and cassava), pulses (soybeans), nuts/seeds (groundnuts), vitamin-A rich fruits and vegetables (mangoes), other fruit (banana and oranges), meat and fish (tilapia and herring), eggs, and other vegetables (eggplant and onion).

Turning to the CoDD indexes, figure 1 presents results for Ghana, showing the price per unit of dietary energy for the lowest cost item in each food group. The lowest-cost foods are usually one of the starchy staples (either maize or cassava), but for several months in 2009 and early 2010 the lowest-cost calorie source was actually from the pulse group (soybeans). The third least expensive source is from the nut group (groundnuts), followed by vitamin A-rich vegetables and fruits (mangoes), and other fruits (bananas). Occasionally, some form of fish (salted dried tilapia or smoked herrings) becomes the fifth group. The cost of reaching the MDD-W is shown by the solid line tracing the price of including that fifth group (CoDD1). An alternative measure showing the average cost of including any group (CoDD2), shown by the dashed line, is higher due to inclusion of the most expensive food groups. A similar analysis in terms of cost per unit of weight, shown in the online supplementary material figure A1, yields qualitatively similar results, except that the “other vegetable” group (represented here by eggplants and onions) becomes cheaper than groundnuts due to its higher moisture content.

**Figure 2:**
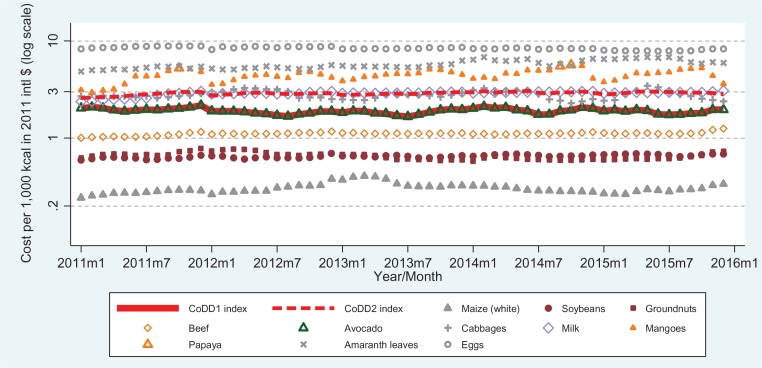
Cost of diet diversity in Tanzania, January 2011–December 2015. *Note:* Foods shown are the least-cost item in their food group that month, as defined by the minimum dietary diversity for women (MDD-W) indicator. Items are ranked in cost per unit of dietary energy. CoDD1 is the cost of reaching the fifth group, and CoDD2 is the cost of including all groups. Groups in ascending order of usual cost are a starchy staple (maize), pulses (soybeans), nuts/seeds (groundnuts), meat or fish (beef for stew), other fruit (avocado), other vegetable (cabbage), dairy (milk), vitamin-A rich fruit (mangoes or papaya), green leafy vegetables (amaranth), and eggs.

Results for CoDD in Tanzania are presented in figure 2, showing that the lowest-cost food group per unit of dietary energy is always the starchy staple (maize), with a pulse (soybean) and a nut (groundnuts) alternating as the second and third least-costly food group, followed by a meat (beef) and a food from the “other fruit” group (avocado). This figure reveals much more stability among the lower-cost food groups than among these foods in Ghana or relative to more expensive food groups in Tanzania. Such differences could reflect the type of market at which food prices are collected, as NBS in Tanzania aims to collect price data for inflation monitoring from the same sellers every time primarily in towns and cities, whereas MoFA in Ghana aims to collect price data for market information purposes from different sellers every time, in a wider variety and greater number of locations. In Tanzania, the relative cost of foods by unit of weight as shown in the online supplementary material figure A2 differs greatly from cost per unit of energy, due to the inclusion of foods with high moisture content notably cabbage, amaranth leaves and milk.

**Figure 3:**
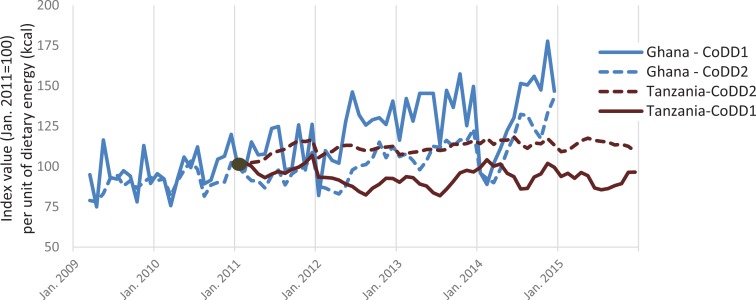
Cost of diet diversity indexes for Ghana and Tanzania, 2009–2015 (January 2011 = 100). *Source:* Index values shown represent changes in the cost of meeting energy needs from diverse food groups, when using the lowest-priced food in each food group as defined for the minimum dietary diversity for women (MDD-W) indicator. CoDD1 shows the cost of reaching five food groups, and CoDD2 shows the average cost of including all available groups. Which foods and food groups are included in this minimally diverse diet varies over time and space. Data shown here are relative to Jan. 2011 prices in real USD/kcal.

To compare changes over time in the marginal cost of obtaining any amount of dietary energy from the fifth food group (CoDD1) or the average of all food groups (CoDD2), it is convenient to standardize costs as index numbers. Results shown in figure 3 reveal differences relative to the base period of January 2011, after which CoDD1 rose sharply with wide swings in Ghana and changed much less in Tanzania. Using all food groups instead of just the fifth, CoDD2 rose less than CoDD1 in Ghana but more in Tanzania, due to differences in price trends among the most and least expensive foods. A similar chart based on cost per unit of weight is provided in our online supplementary material figure A3, revealing qualitatively similar results in most months. Focusing on our preferred cost of dietary diversity measure in figure 3, CoDD1 per unit of dietary energy rose by about 50% from January 2011 to late 2014, while in Tanzania, the price indices were relatively stable from January 2011 to December 2015.

We can compare the cost of dietary diversity by food group to the cost of nutrient adequacy (CoNA) using each month’s solution to equations (3) – (6). For Ghana, a total of eight distinct food items are ever included in those least-cost diets. Three of these foods (mangoes, soybeans and smoked herring) are included every month. Mangoes and soybeans enter with mean intakes of 900 and 256 g/day respectively, as they are the principal sources of limiting nutrients which are more costly to obtain from other sources in the Ghanaian context. Such a high level of consumption for these two foods is not realistic or recommended, but does reveal the degree to which the nutrient profile of mango and soybean fills gaps left by other foods listed in table 3 below.

**Table 3:** Foods Selected for CoNA Diet Plans in Ghana, Mar 2009–Dec 2014

Food Item	2009–2014		2009		2010		2011		2012		2013		2014	
	Mean	% Selected	Mean	% Selected	Mean	% Selected	Mean	% Selected	Mean	% Selected	Mean	% Selected	Mean	% Selected
Cassava	21	11%	18	10%	–	–	47	25%	63	33%	–	–	–	–
Maize	50	69%	14	20%	48	67%	55	75%	36	50%	66	92%	74	100%
Mangoes	900	100%	910	100%	904	100%	902	100%	905	100%	881	100%	899	100%
Paddy Rice	14	49%	–	–	6	25%	18	67%	13	50%	15	42%	27	100%
Palm Oil	4	51%	7	100%	6	75%	3	33%	4	50%	5	58%	–	–
Plantain	3	1%	–	–	–	–	–	–	–	–	19	8%	–	–
Smoked Herrings	15	100%	15	100%	15	100%	15	100%	15	100%	15	100%	15	100%
Soybeans	256	100%	289	100%	267	100%	242	100%	252	100%	246	100%	243	100%

*Note:* Data shown are mean intake (g/day) and intake frequency (percent of days) for lowest-cost diets that reach the estimated average requirement (EAR) of essential nutrients for an adult woman of 55 kg at an energy level of 2,000 kcal/day.

**Figure 4:**
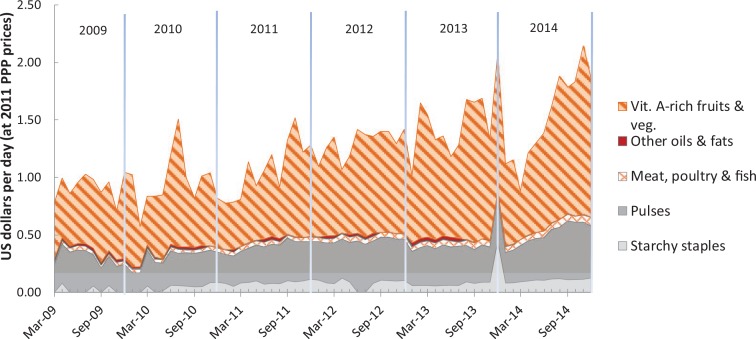
Cost of nutrient adequacy by food group in Ghana, Mar 2009–Dec 2014. *Note:* Data shown are total cost in each month of the foods needed for lowest cost of nutrient adequacy (CoNA), for an adult woman of 55kg at a dietary energy level of 2,000 kcal/day.

As shown in figure 4, the CoNA index for Ghana more than doubled from USD 0.78 per day in March 2009 to USD 1.87 in December 2014. We can link the foods that account for this rise back to the food groups used for CoDD, noting that mangoes from the vitamin A-rich fruits and vegetables group accounted for more than 60% of CoNA on average. Soybeans from the pulses group contributed about 28% of CoNA on average, while cassava from the starchy staples group, and smoke herrings from the flesh-foods group accounted for approximately 6% and 4%, respectively. The remaining cost was palm oil, which is not included in CoDD and which contributed about 1.5% of CoNA before July 2013, then not selected for least-cost diet packages thereafter.

**Figure 5:**
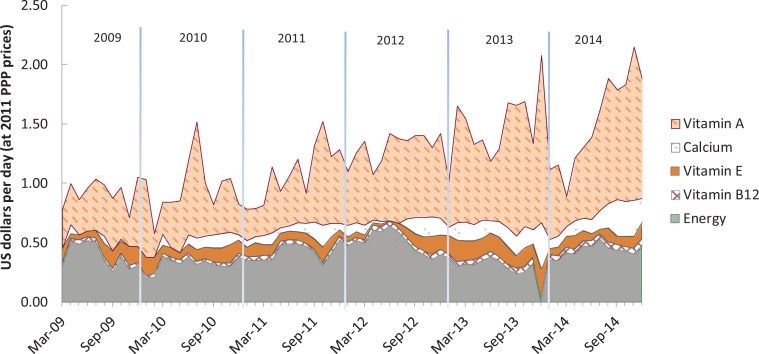
Cost of nutrient adequacy by limiting nutrient in Ghana, Mar 2009–Dec 2014. *Note:* Data shown are total cost in each month of the foods needed for lowest cost of nutrient adequacy (CoNA), for an adult woman of 55 kg at a dietary energy level of 2,000 kcal/day.

As shown in table 4, in Ghana a total of five nutrients have limiting EARs, four of which were limiting nutrients in all months. Vitamin A, as the most expensive nutrient, has a shadow price elasticity (SPE) of 0.47, meaning that CoNA increases by 0.47% when the EAR for vitamin A increases by 1%, that is, from 500 mcg to 505 mcg per day. Dietary energy is still a very important constraint in Ghana with an average SPE of 0.34. As shown in figure 5, the nutrients that are most limiting for CoNA in Ghana are vitamin A, followed by dietary energy, vitamin E, calcium and vitamin B12.

**Table 4:** Nutrient Requirements Contributing to CoNA in Ghana, Mar 2009–Dec 2014

Nutrient	2009–2014		2009		2010		2011		2012		2013		2014	
	%EAR	SPE	%EAR	SPE	%EAR	SPE	%EAR	SPE	%EAR	SPE	%EAR	SPE	%EAR	SPE
Always limiting nutrients														
Energy	100%	0.344	100%	0.423	100%	0.344	100%	0.402	100%	0.391	100%	0.213	100%	0.302
Vitamin B12	100%	0.029	100%	0.032	100%	0.029	100%	0.030	100%	0.027	100%	0.027	100%	0.032
Vitamin A	100%	0.467	100%	0.420	100%	0.448	100%	0.407	100%	0.470	100%	0.548	100%	0.500
Vitamin E	100%	0.086	100%	0.109	100%	0.116	100%	0.082	100%	0.049	100%	0.107	100%	0.058
Sometimes limiting nutrients														
Calcium	104%	0.074	114%	0.016	107%	0.063	100%	0.079	103%	0.062	100%	0.104	100%	0.109

*Note:* Data shown are mean fraction of the estimated average requirement for an adult woman of 55 kg at an energy level of 2,000 kcal/day consumed each day (%EAR). The mean shadow price elasticity (SPE) of each nutrient when it is limiting. SPE is defined as the percentage change of CoNA if the EAR for that nutrient were increased by 1%.

For Tanzania, the CoNA solution to equations (3) – (6) spans sixty months from January 2011 to December 2015. As shown in table 5, a total of eight food items are ever selected, of which two (white maize and mchicha or amaranth leaves) are included in every month with mean intakes of 255 and 197 g/d, respectively.

**Table 5:** Foods Consumed to Construct CoNA Diet Plans in Tanzania, Jan 2011–Dec 2015

Food Item	2011–2015		2011		2012		2013		2014		2015	
	Mean g/day	Pct. of months	Mean g/day	Pct. of months	Mean g/day	Pct. of months	Mean g/day	Pct. of months	Mean g/day	Pct. of months	Mean g/day	Pct. of months
Cabbages	25	80%	14	33%	24	67%	34	100%	26	100%	27	100%
Cassava flour	0.1	7%	–	–	1	33%	–	–	–	–	–	–
Cooking oil	9.2	32%	29	100%	12	42%	–	–	–	–	4	17%
Dried sardines	14	100%	14	100%	14	100%	14	100%	14	100%	14	100%
Mchicha (amaranth)	197	100%	263	100%	203	100%	143	100%	188	100%	187	100%
Groundnuts	89	68%	–	–	74	58%	127	100%	131	100%	111	83%
Soybeans	92	78%	69	100%	91	92%	122	83%	85	58%	92	58%
White Maize	255	100%	369	100%	269	100%	183	100%	219	100%	232	100%

*Note:* Data shown are mean intake (g/day) and intake frequency (percent of days) for lowest-cost diets that reach the estimated average requirement (EAR) of essential nutrients for an adult woman of 55 kg at an energy level of 2,000 kcal/day.

**Figure 6:**
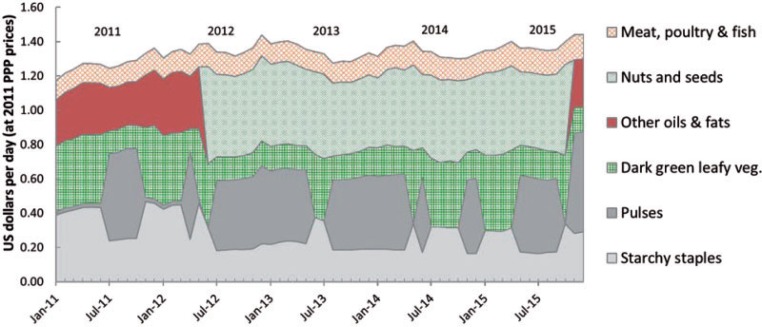
Cost of nutrient adequacy by food group in Tanzania, Jan 2011–Dec 2015. *Note:* Data shown are total cost in each month of the foods needed for lowest cost of nutrient adequacy (CoNA), for an adult woman of 55 kg at a dietary energy level of 2,000 kcal/day.

Figure 6 reveals that the CoNA indicator for Tanzania fluctuated much less than for Ghana, rising gradually by about 25%, from 1.17 to 1.48 USD/day over these five years, but there is sharp variation in the composition of this least-cost diet over time. Pulses enter periodically when soy is relatively inexpensive, displacing both the lowest-cost starchy staple (maize) and the lowest-cost green leafy vegetable (mchicha), and cooking oil is displaced by nuts and seeds (groundnuts). A dried fish (sardines) remains in this least-cost diet at about 10% of its total cost throughout the period.

**Figure 7:**
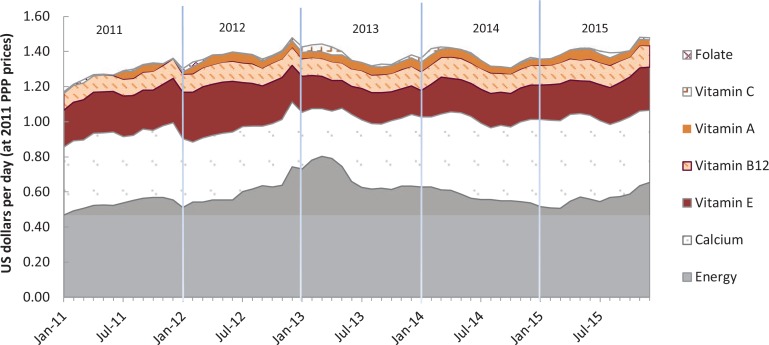
Cost of nutrient adequacy by limiting nutrient in Tanzania, Jan 2011–Dec 2015. *Note:* Data shown are total cost in each month of the foods needed for lowest cost of nutrient adequacy (CoNA), for an adult woman of 55 kg at a dietary energy level of 2,000 kcal/day.

In Tanzania, as shown in table 6 there were in total of seven limiting nutrients, including the same five limiting nutrients as Ghana plus vitamin C and selenium. Dietary energy, calcium, vitamin C, B12 and E were limiting nutrients in all observations. Using the SPE as a criterion, dietary energy was the most constraining nutritional factor in Tanzania, as a 1% increase in daily dietary energy requirement from 2,000 to 2,020 kcal would increase CoNA by 0.4%. The most constraining individual nutrient was calcium with an average SPE of 0.3, meaning an increase in CoNA of 0.3% if calcium requirements rose from 800 mg to 808 mg. Vitamin A is less costly in Tanzania than in Ghana, with an SPE of only 0.027. As shown in figure 7, energy became increasingly constraining until early 2013, and then calcium became relatively more important until early 2015 when the relative cost of acquiring dietary energy rose again.

**Table 6:** Nutrient Requirements Contributing to CoNA in Tanzania, Jan 2011–Dec 2015

Nutrient	2011–2015		2011		2012		2013		2014		2015	
	% EAR	SPE	% EAR	SPE	% EAR	SPE	% EAR	SPE	% EAR	SPE	% EAR	SPE
Always limiting nutrients												
Energy	100%	0.433	100%	0.415	100%	0.430	100%	0.498	100%	0.422	100%	0.401
Calcium	100%	0.296	100%	0.312	100%	0.266	100%	0.254	100%	0.318	100%	0.327
Vitamin E	100%	0.157	100%	0.179	100%	0.189	100%	0.127	100%	0.139	100%	0.150
Vitamin B12	100%	0.080	100%	0.077	100%	0.079	100%	0.075	100%	0.083	100%	0.031
Sometimes limiting nutrients												
Vitamin A	100%	0.027	100%	0.011	100%	0.030	100%	0.031	100%	0.032	100%	0.031
Vitamin C	154%	0.006	198%	0.001	158%	0.003	119%	0.014	147%	0.006	147%	0.005
Folate	228%	0.001	151%	0.003	218%	0.004	278%	–	250%	–	243%	–

*Note:* Data shown are mean fraction of the estimated average requirement for an adult woman of 55 kg at an energy level of 2,000 kcal/day consumed each day (%EAR). The mean Shadow Price Elasticity (SPE) of each nutrient when it is limiting. SPE is defined as the percentage change of CoNA if the EAR for that nutrient were increased by 1%.

## Discussion and Conclusions

This paper presents nutritional price indexes to compare the relative cost of reaching international nutrition standards at each time and place. We introduce a cost of diet diversity (CoDD) index, defined as the minimum cost of acquiring at least five out of ten specific food groups, for comparison with the cost of nutrient adequacy (CoNA), which tracks the minimum cost of meeting estimated average requirements of energy, protein and seventeen essential nutrients. These indexes reveal temporal and spatial differences in access to diverse diets and adequate nutrients, helping to guide policies and programs aimed at improving their affordability in local markets.

Using national average monthly prices for Ghana from 2009 through 2014, we find that the cost of meeting the diet diversity standard fluctuated seasonally and rose sharply from mid-2010 through 2014 at about 10% per year faster than inflation, due primarily to rising relative prices for fruit. The cost of nutrient adequacy doubled over this period, due primarily to increased cost of foods needed to meet standards for vitamin A and also calcium. Similar monthly data for Tanzania show an upward trend from 2011 to 2012 and then seasonal fluctuations through 2015, switching among different leguminous grains and green leafy vegetables as the lowest-cost way to meet nutrient needs. In both Ghana and Tanzania, vitamin B-12 needs lead to inclusion of dried fish in CoNA indexes, even though it is not included for diet diversity purposes in CoDD. The cost of meeting nutrient needs in both countries is also heavily driven by daily energy requirements, with each 1% rise in energy intake leading to an 0.3–0.5% rise in the least-cost diet. Comparing results for CoDD and CoNA reveals the continued importance of year-round access to basic staple foods for macronutrients, while identifying the other foods and specific micronutrients that limit access to healthier diets.

The CoDD and CoNA indexes are intended to track access and affordability of foods required for a given nutritional standard, which may be very different from what is actually consumed. Actual diets often fall short of international standards for some nutrients, while exceeding minimal needs in other dimensions, driven in part by relative prices among different foods. CoDD is a unit-free measure to track changes in relative prices, while CoNA is a cost per day which we can compare to income levels or actual expenditure. In 2012, for example, the level of CoNA in both Ghana and Tanzania was about $1.40 per person at 2011 PPP prices, while national average per-capita food expenditure in rural areas was estimated at $1.73 in Tanzania and $2.99 in Ghana (IFPRI 2017). Low incomes make nutritious diets out of reach for many people, especially in Tanzania, but even when incomes are higher as in Ghana the relative cost of different foods will influence food choice. CoNA is particularly useful for identifying foods such as soybeans that have recently become low-cost sources of essential nutrients, and might therefore play an increasing role in local diets as culinary practices evolve.

Nutritional price indexes like CoDD and CoNA can guide public investment, policies and programs that make high-quality diets more accessible year-round even in remote areas, complementing farmers’ self-provisioning with interventions that lower the relative cost of nutrient-dense foods on local markets. Introducing these indexes could increase demand for price data about a wider range of nutritious foods at various places. This paper uses two very different data sources: the Ministry of Food and Agriculture (MoFA) market information service in Ghana, and the National Bureau of Statistics system for monitoring inflation in Tanzania. The Ghana data are intended to inform production and marketing of traded crops, and historically omitted two nutritionally important kinds of food: dark green leafy vegetables and dairy. Demand for data to construct CoDD and CoNA has already led MoFA to expand their market information service to a wider range of foods (Nortey 2017), with preliminary analysis of the new data by the World Food Program revealing additional opportunities to meet nutritional targets at lower cost beyond the results in this paper (WFP 2017). Using CoDD and CoNA can also guide statistical agencies to collect price data at times and places when nutritious diets are most out of reach. For example, ongoing studies using the Tanzania data identify regional differences in seasonality (Bai et al. 2018), revealing where and when price fluctuations are most important to measure and eventually address with investments in market infrastructure tailored to the specific foods needed for more nutritious diets.

Our CoDD index aims to guide interventions that target diet diversity, in this case to reach the MDD-W threshold of at least five food groups from a list of ten. This is useful for settings where diversity as such is important (Clements and Si 2018), across specific food groups as in the U.S. Healthy Eating Index used by Beatty, Lin, and Smith (2014) for the United States. In developing countries, many agencies aim explicitly to increase the proportion of women whose diets meet the MDD-W threshold (e.g., Feed the Future 2018), and the CoDD price index can reveal which foods drive the cost of reaching that goal at each time and place. CoDD can also inform where and when the cost of reaching MDD-W is highest, to help target interventions towards more universal access to adequate dietary diversity.

The CoNA index adds information about the quantities of each food needed to meet nutritional targets, identifying which nutrients are most expensive and which foods are most-effective at each time and place. Our results reveal the universal importance of a few staple foods to meet macronutrient constraints in both Ghana and Tanzania, even as a variety of other foods are also needed to reach micronutrient standards at different locations and times of year. Fruits and vegetables have not traditionally been prioritized for either public investment or data collection, but CoNA suggests that they often play an outsized role in the cost of nutritious diets. Using CoNA can guide interventions toward the lowest-cost way to achieve many aspects of food security targeted by international agreements (FAO 1996) and reveal which nutrients remain most difficult to obtain even after policies and programs attempt to expand food access. For those nutrients, fortification and supplementation are important options, as shown by an analysis of our data regarding opportunities for staple flour fortification in Ghana (WFP 2017).

In summary, the index proposed here for the cost of diet diversity, alongside traditional measures for the cost of nutrient adequacy, allow us to measure changes in the (un)affordability of healthier diets than those currently consumed. Monitoring changes in these indexes can reveal the degree to which policy and program interventions improve access to nutritious diets, focusing on the needs of low-income people who seek the least expensive foods at each time and place. One key limitation of the work so far is that existing price monitoring systems often miss foods of nutritional importance for the poor, and even when prices are available their nutritional content may not have been measured. Future research using available and new data can identify which of the functional forms discussed here are most sensitive to differences in local food environments, and most predictive of differences in nutrition outcomes. Formulation of the indexes could also spur improvements in data collection systems, to include all locally available foods that might help meet nutritional standards at prices that accurately reflect the cost of acquisition for local households. More complete data collection would, in turn, improve the value of new indexes, to analyze how specific policies and programs alter the cost of meeting international standards for a nutritious diet.
