# Supplementary material for: Measuring the Affordability of Nutritious Diets in Africa: Price Indexes for Diet Diversity and the Cost of Nutrient Adequacy
Source: Am J Agric Econ. 2018 Aug 13;100(5):1285–301. doi: 10.1093/ajae/aay059 (PMC7053386; doi:10.1093/ajae/aay059)
Supplement: Supplementary file 1 [file AJAE-2018-AJAE-AAY059-S1.pdf]

Annex of Supplemental Information for the American Journal of Agricultural Economics

Measuring the Affordability of Nutritious Diets in Africa: Price Indexes for Diet Diversity and the Cost of Nutrient Adequacy

William A. Masters[1,2], Yan Bai[1], Anna Herforth[1], Daniel Sarpong[3], Fulgence Mishili[4], Joyce Kinabo[5] and Jennifer C. Coates[1]

This version revised 20 June 2018

Author affiliations:

1. Friedman School of Nutrition Science & Policy, Tufts University, USA
2. Department of Economics, Tufts University, USA
3. Dept. of Agricultural Economics & Agribusiness, University of Ghana
4. Dept. of Agricultural Economics & Agribusiness, Sokoine University of Agriculture, Tanzania
5. Dept. of Food Technology, Nutrition & Consumer Sciences, Sokoine Univ. of Agric., Tanzania

Note:

This annex is supplemental information accompanying the AJAE article entitled "Measuring the Affordability of Nutritious Diets in Africa: Price Indexes for Diet Diversity and the Cost of Nutrient Adequacy". Data and model code for replication of results is available at our project website, http://sites.tufts.edu/candasa.

Table A1. Descriptive Statistics of Monthly Food Prices per kg – Ghana (2011$)

Food Groups	No	Foodstuffs	Obs.	Mean	Std. Dev.	CV	Min	Max
**Grains, white roots and tubers, and plantains**	1	Cassava	70	0.53	0.11	0.20	0.36	0.76
	2	Cocoyam	70	1.19	0.27	0.23	0.79	1.82
	3	Kokonte	70	1.27	0.21	0.17	0.90	1.81
	4	Gari	70	1.47	0.25	0.17	1.15	2.41
	5	Imported Rice	70	2.68	0.42	0.16	2.19	3.98
	6	Local Rice	70	1.86	0.22	0.12	1.51	2.67
	7	Maize	70	0.95	0.17	0.18	0.69	1.44
	8	Millet	70	1.48	0.19	0.13	1.16	1.94
	9	Paddy Rice	56	1.27	0.40	0.32	0.76	2.73
	10	Plantains	70	1.79	0.60	0.33	1.11	4.12
	11	Sorghum	70	1.20	0.13	0.11	0.97	1.53
	12	Yam	70	1.23	0.20	0.16	0.90	1.75
**Pulses**	13	Cowpea	70	2.06	0.35	0.17	1.45	2.84
	14	Soybeans	70	1.30	0.31	0.24	0.57	2.11
**Nuts & seeds**	15	Groundnut	70	3.26	0.62	0.19	2.25	4.45
**Meat, poultry and fish**	16	Anchovies	70	6.33	1.37	0.22	3.19	11.68
	17	Salted Dried Tilapia Fish	70	2.43	0.58	0.24	0.99	4.15
	18	Smoked Herrings	70	3.15	0.70	0.22	2.01	5.45
**Eggs**	19	Eggs	70	8.90	0.63	0.07	7.47	10.84
**Vitamin A-rich vegetables and fruits**	20	Mangoes	70	0.85	0.31	0.36	0.38	1.76
**Other vegetables**	21	Garden Eggs	70	2.29	0.59	0.26	1.20	4.14
	22	Large Onions	70	3.58	1.16	0.32	1.68	5.81
	23	Tomatoes	70	3.74	1.24	0.33	1.82	7.18
**Other fruits**	24	Bananas	70	1.69	0.33	0.20	1.02	2.53
	25	Oranges	70	1.35	0.41	0.31	0.55	3.09
	26	Pineapples	70	1.47	0.16	0.11	1.15	1.94

Note: Authors' calculations, from Ghana Ministry of Food and Agriculture data. Two food groups in the MDD-W are not represented in this dataset: Dairy, and Dark Green Leafy Vegetables. Kokonte and Gari refer to processed cassava products. Groundnut refers to shelled groundnut, with prices for unshelled groundnut omitted from data analysis.

Table A2. Descriptive Statistics of Monthly Food Prices per kg – Tanzania (2011$)

Food Group	No	Foodstuff	Obs.	Mean	Std. Dev.	CV	Min	Max
**Grains, white roots and tubers, and plantains**	1	Cassava flour	60	2.00	0.23	0.11	1.62	2.63
	2	Cassava fresh	60	1.23	0.11	0.09	0.95	1.43
	3	Cooking Bananas Green	60	2.00	0.11	0.05	1.77	2.32
	4	Finger millet	60	2.55	0.43	0.17	1.89	3.30
	5	Maize Flour	60	1.69	0.20	0.12	1.32	2.28
	6	Potatoes – round	60	1.73	0.10	0.06	1.52	2.02
	7	Rice	60	2.68	0.42	0.16	2.07	3.53
	8	Sweet Potatoes	60	1.47	0.12	0.08	1.25	1.69
	9	Wheat Flour	60	2.24	0.14	0.06	2.03	2.57
	10	White Maize	60	1.11	0.13	0.12	0.89	1.49
**Pulses**	11	Soybeans	60	2.90	0.11	0.04	2.65	3.10
	12	Lentils	60	3.81	0.35	0.09	3.20	4.40
	13	Red dry beans	60	2.63	0.13	0.05	2.43	2.94
**Nuts & seeds**	14	Natural Groundnuts	60	3.72	0.31	0.08	3.28	4.44
**Dairy**	15	Fresh cow milk	60	1.76	0.10	0.05	1.45	1.87
	16	Powdered milk	60	39.56	1.86	0.05	34.77	43.18
**Meat, poultry and fish**	17	Beef sausage	60	17.48	0.34	0.02	16.95	18.37
	18	Beef with bones	60	10.89	0.52	0.05	9.65	12.32
	19	Beef without bones	60	10.89	0.40	0.04	9.87	12.30
	20	Dried sardines	60	8.99	0.70	0.08	7.68	10.37
	21	Goat meat	60	10.37	0.41	0.04	9.12	11.10
	22	Industrially bred live chicken	60	13.99	0.67	0.05	11.92	14.88
	23	Pork meat	60	11.93	1.04	0.09	9.22	13.63
	24	Traditionally bred live chicken	60	25.34	1.69	0.07	21.16	28.23
**Eggs**	25	Eggs-layers	60	12.05	0.41	0.03	11.28	12.70
	26	Eggs-traditional	60	16.89	0.99	0.06	14.73	18.11
**Dark green leafy vegetables**	27	Mchicha (spinach)	60	1.72	0.17	0.10	1.46	2.05
**Vitamin A-rich vegetables and fruits**	28	Carrots	60	2.89	0.28	0.10	2.46	3.72
	29	Mangoes	60	2.67	0.38	0.14	1.78	3.64
	30	Papaya	60	2.42	0.22	0.09	2.03	2.86

Table A2 (continued)

Food Group	No	Foodstuff	Obs.	Mean	Std. Dev.	CV	Min	Max
**Other vegetables**	31	Bitter tomatoes	60	2.22	0.12	0.05	1.96	2.68
	32	Egg plant	60	2.36	0.12	0.05	2.12	2.71
	33	Cabbages	60	1.15	0.11	0.10	0.94	1.43
	34	Green peas	60	20.07	1.41	0.07	16.78	23.01
	35	Green bell pepper	60	3.29	0.18	0.06	2.96	3.83
	36	Ladies finger (okra)	60	3.72	0.25	0.07	3.29	4.37
	37	Onions	60	2.57	0.31	0.12	2.08	3.54
	38	Tomatoes red	60	1.88	0.21	0.11	1.50	2.44
**Other fruits**	39	Apples (Imported)	60	10.18	0.84	0.08	8.24	12.28
	40	Avocado	60	3.05	0.19	0.06	2.67	3.49
	41	Coconut mature	60	8.94	0.83	0.09	7.75	11.10
	42	Lemons	60	3.41	0.59	0.17	2.39	5.22
	43	Limes	60	4.69	0.86	0.18	3.60	7.07
	44	Oranges	60	2.04	0.21	0.10	1.60	2.59
	45	Pineapples	60	3.33	0.33	0.10	2.77	3.99
	46	Sweet banana	60	2.98	0.25	0.08	2.41	3.48

Note: Authors' calculations, from Tanzania Bureau of Statistics data.

Figure A1. Cost of diet diversity in Ghana per unit of weight, March 2009-December 2014

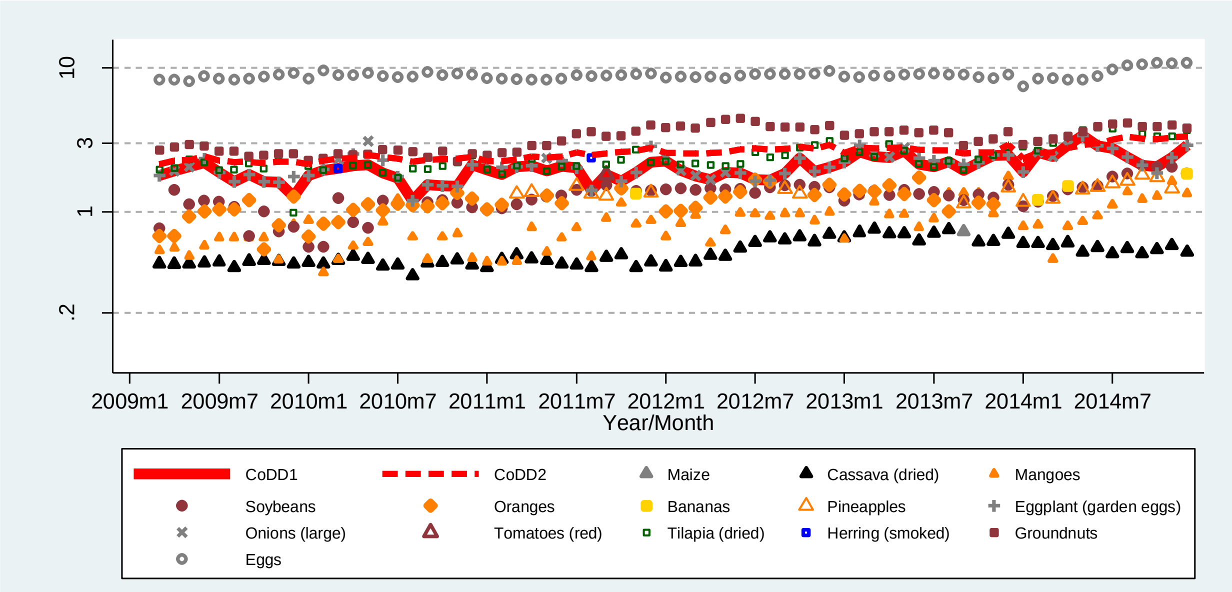

Note: Foods shown are the least-cost item in their food group that month, as defined by the Minimum Dietary Diversity for Women (MDD-W) indicator. Items are ranked in cost per kilogram. CoDD1 is the cost of reaching the 5th group, and CoDD2 is the cost of including all groups. Groups in ascending order of usual cost are starchy staples (cassava & maize), pulses (soybeans), vitamin-A rich fruits and vegetables (mangoes), other fruit (oranges, banana and pineapples), other veg (eggplant, onion, tomatoes), meat/fish (tilapia & herring), nuts/seeds (groundnuts), eggs.

Figure A2. Cost of diet diversity in Tanzania per unit of weight, January 2011 – December 2015

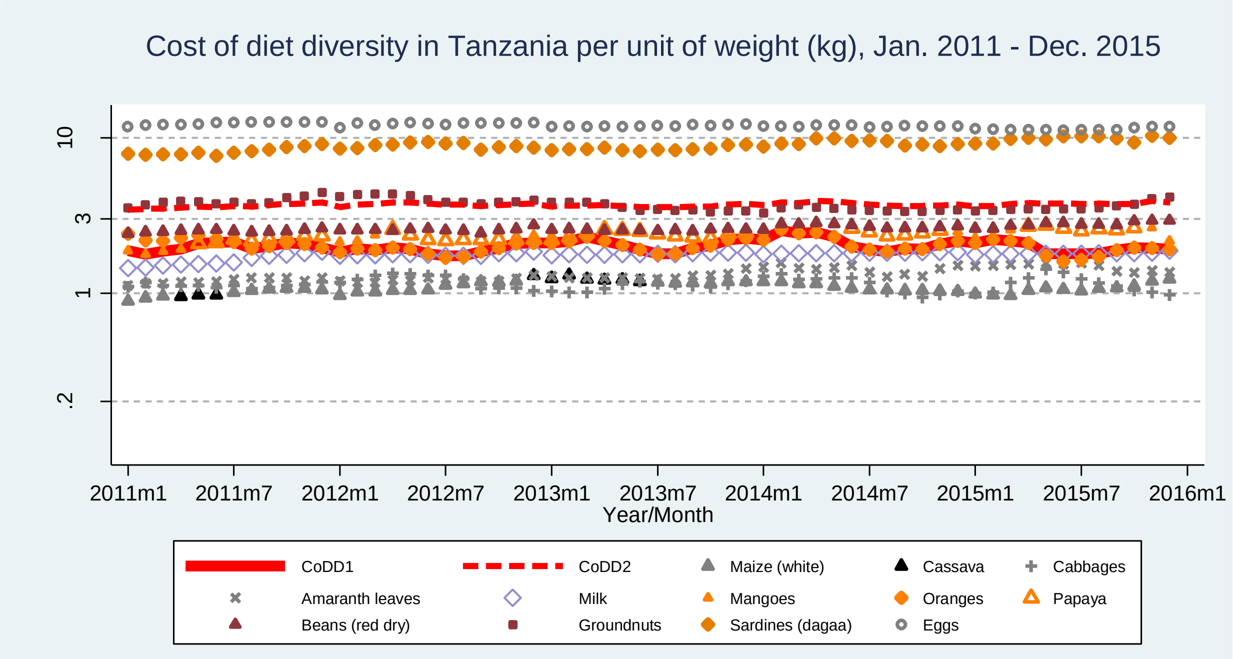

Note: Foods shown are the least-cost item in their food group that month, as defined by the Minimum Dietary Diversity for Women (MDD-W) indicator. Items are ranked in cost per kilogram. CoDD1 is the cost of reaching the 5th group, and CoDD2 is the cost of including all groups. Groups in ascending order of usual cost are starchy staples (cassava & maize), other veg. (cabbage), dark green leafy veg. (amaranth leaves), milk, vitamin-A rich fruits and vegetables (mangoes, papaya), other fruit (oranges), pulses (beans), nuts/seeds (groundnuts), meat/fish (sardines), eggs.

Figure A3. Cost of Diet Diversity indexes for Ghana and Tanzania, 2009-2015 (January 2011 = 100)

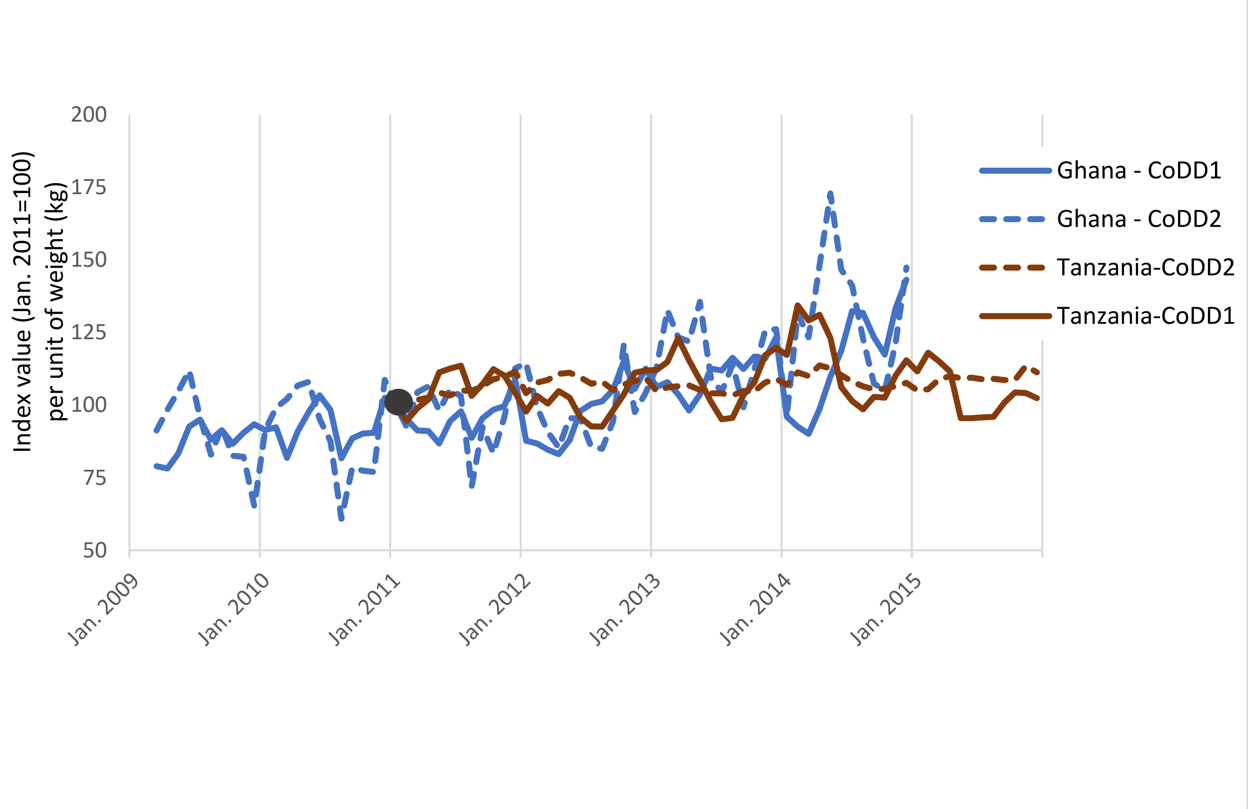

Source: Calculated from Ghana Ministry of Food and Agriculture (MoFA) and Tanzania National Bureau of Statistics (NBS) file data. Index values shown represent changes in the cost of consuming foods from diverse food groups, when using the lowest-priced food in each food group as defined for the Minimum Dietary Diversity for Women (MDD-W) indicator. CoDD1 shows the cost of reaching five food groups, and CoDD2 shows the average cost of including all available groups. Which foods and food groups are included in this minimally diverse diet varies over time and space. Data shown here are relative to January 2011 prices in real USD per kg.

Figure A4. Cost of Diet Diversity (CoDD) and Cost of Nutrient Adequacy (CoNA) indexes for Ghana and Tanzania, 2009-2015 (January 2011 = 100)

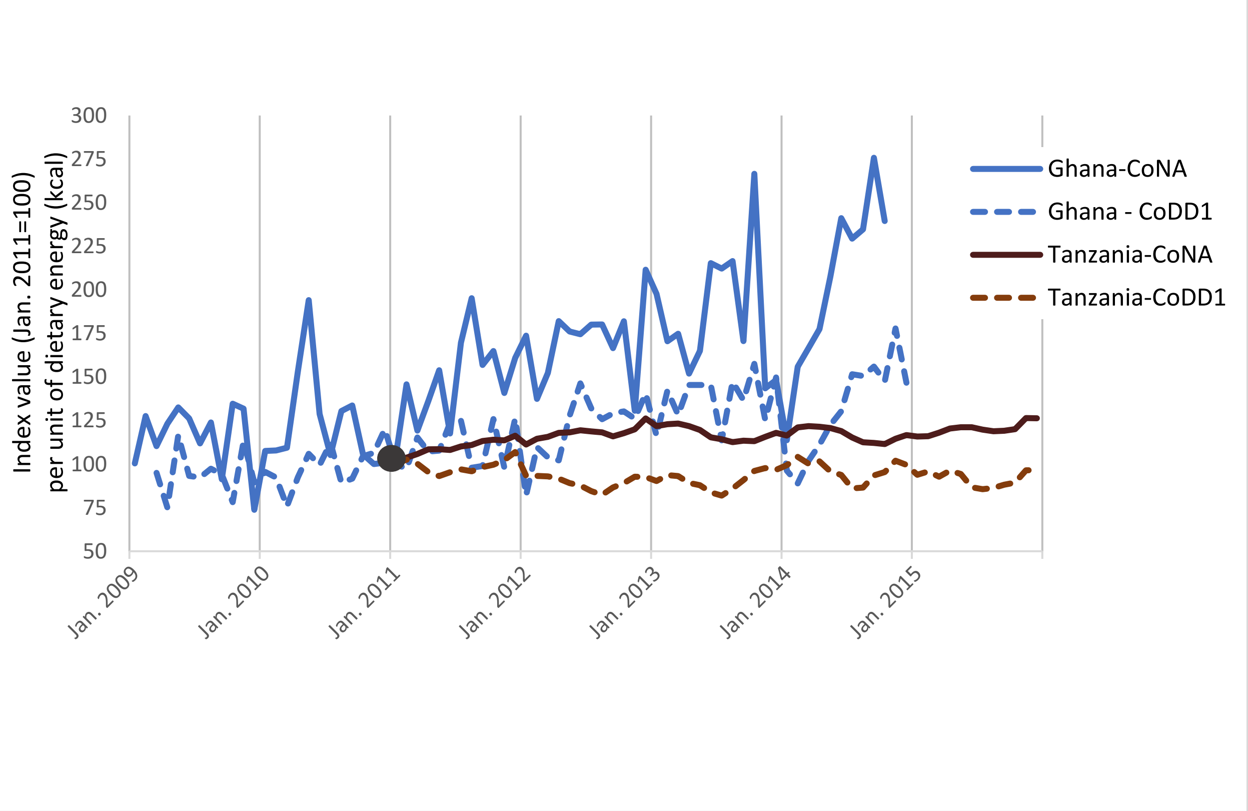

Source: Calculated from Ghana Ministry of Food and Agriculture (MoFA) and Tanzania National Bureau of Statistics (NBS) file data. Index values shown represent changes in the cost of meeting energy needs from diverse food groups (CoDD1) and reaching nutrient adequacy (CoNA). Which foods and food groups are included in the index varies over time and space. Data shown here are relative to Jan. 2011 prices in real USD/kcal.

Figure A5. The Cost of Nutrient Adequacy (CoNA) in Ghana and Tanzania, 2009-15

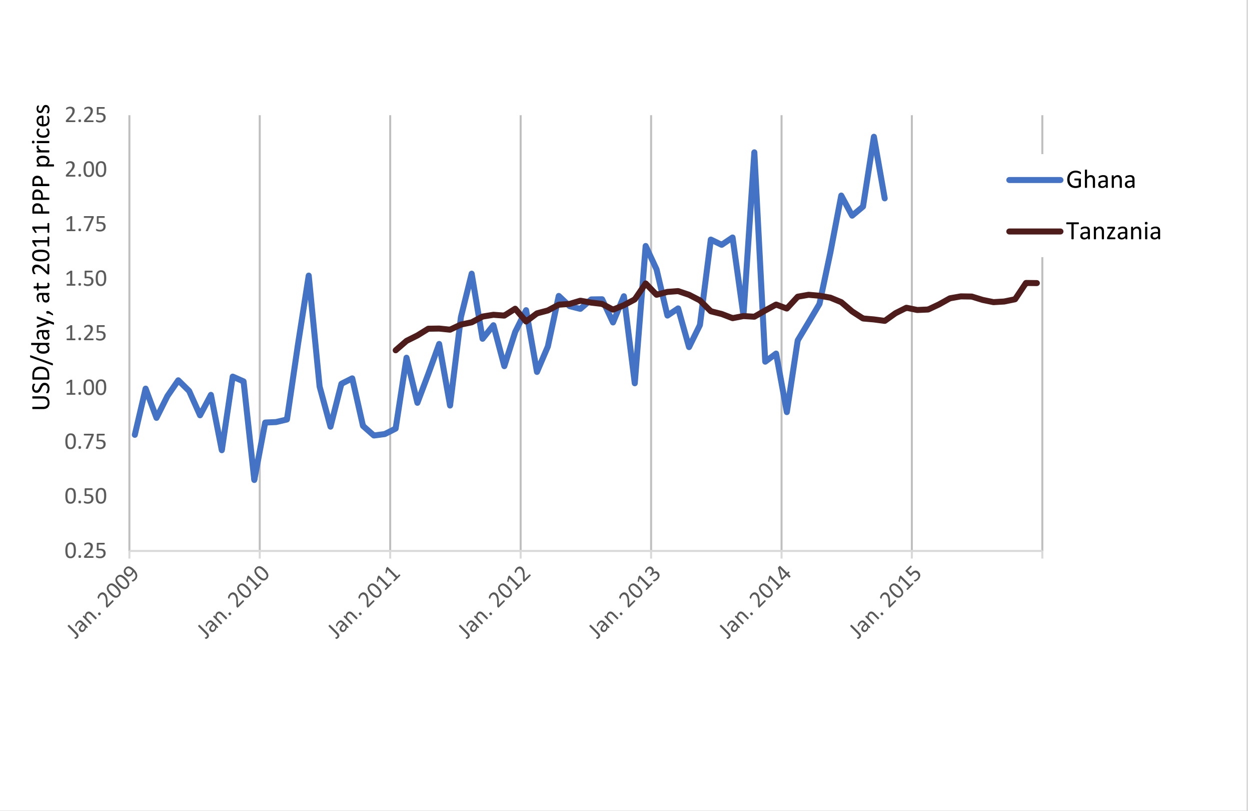

Source: Calculated from Ghana Ministry of Food and Agriculture (MoFA) and Tanzania National Bureau of Statistics (NBS) file data. Index values shown represent the value per day of a least-cost diet, meeting daily needs for dietary energy, protein and 17 micronutrients based on estimated average requirements for an adult woman. Foods included in the least-cost diet vary over time and space, providing a lower bound on the cost of meeting nutrient needs given other goals such as food preferences.

Table A3. Nutritional and Dietary Energy Criteria

No	Nutrient Groups	Nutrients	EARs [1]	Units
1	**Dietary Energy**	Energy	2,000	kcal/day
2	**Macronutrient**	Protein [2]	36.3	g/day
3	**Minerals**	Calcium	800	mg/day
4		Iron	8.1	mg/day
5		Magnesium	255	mg/day
6		Phosphorus	580	mg/day
7		Zinc	6.8	mg/day
8		Copper	0.7	mcg/day
9		Selenium	45	mcg/day
10	**Vitamins**	Vitamin C	60	mg/day
11		Thiamin	0.9	mg/day
12		Riboflavin	0.9	mg/day
13		Niacin	11	mg/day
14		Vitamin B6	1.1	mg/day
15		Folate	320	mg/day
16		Vitamin B12	2	mcg/day
17		Vitamin A	500	mcg/day
18		Vitamin E	12	mg/day

Note: Data shown are estimated Average Requirement (EAR) for adult women from 19 to 30 years old, from Dietary Reference Intakes (DRIs) developed by the U.S. Institute of Medicine of the National Academies. EAR of protein is calculated based on an adult woman of 55kg at a dietary energy level of 2000 kcal/day.

Table A4. Food Items, Food Groups and Food Composition (Ghana)

Foods	Food Groups	Energy	Protein	Calcium	Iron	Magnesium	Phosphorus	Zinc	Vitamin C	Thiamin	Riboflavin	Niacin	Vitamin B6	Folate	Vitamin B12	Vitamin A	Vitamin E	Copper	Selenium
**ANCHOVIES**	Meat, poultry and fish	1.310	0.204	1.470	0.033	0.410	1.740	0.017	0.000	0.001	0.003	0.140	0.001	0.090	0.006	0.150	0.006	0.002	0.365
**BANANA**	Other fruits	0.890	0.011	0.050	0.003	0.270	0.220	0.002	0.087	0.000	0.001	0.007	0.004	0.200	0.000	0.030	0.001	0.001	0.010
**CASSAVA**	Grains, white roots and tubers, and plantains	1.600	0.014	0.160	0.003	0.210	0.270	0.003	0.206	0.001	0.000	0.009	0.001	0.270	0.000	0.010	0.002	0.001	0.007
**COCONUT OIL**	Other oils and fats	8.920	0.000	0.010	0.001	0.000	0.000	0.000	0.000	0.000	0.000	0.000	0.000	0.000	0.000	0.000	0.001	0.000	0.000
**COCOYAM**	Grains, white roots and tubers, and plantains	1.120	0.015	0.430	0.006	0.330	0.840	0.002	0.045	0.001	0.000	0.006	0.003	0.220	0.000	0.040	0.024	0.002	0.007
**COWPEA**	Pulses	3.360	0.235	1.100	0.083	1.840	4.240	0.034	0.015	0.009	0.002	0.021	0.004	6.330	0.000	0.030	0.004	0.008	0.090
**DRIED CASSAVA CHIPS (KOKONTE)**	Grains, white roots and tubers, and plantains	3.350	0.019	1.380	0.015	0.450	1.020	0.007	0.040	0.001	0.001	0.012	0.002	0.470	0.000	0.000	0.004	0.002	0.000
**EGGS**	Eggs	1.430	0.126	0.560	0.018	0.120	1.980	0.013	0.000	0.000	0.005	0.001	0.002	0.470	0.009	1.600	0.011	0.001	0.307
**GARDEN EGGS**	Other vegetables	0.250	0.010	0.090	0.002	0.140	0.240	0.002	0.022	0.000	0.000	0.006	0.001	0.220	0.000	0.010	0.003	0.001	0.003
**GARI**	Grains, white roots and tubers, and plantains	3.350	0.019	1.380	0.015	0.450	1.020	0.007	0.040	0.001	0.001	0.012	0.002	0.470	0.000	0.000	0.004	0.002	0.000
**GROUNDNUT**	Nuts and seeds	5.670	0.258	0.920	0.046	1.680	3.760	0.033	0.000	0.006	0.001	0.121	0.003	2.400	0.000	0.000	0.083	0.011	0.072
**GROUNDNUT OIL**	Other oils and fats	8.840	0.000	0.000	0.000	0.000	0.000	0.000	0.000	0.000	0.000	0.000	0.000	0.000	0.000	0.000	0.157	0.000	0.000
**IMPORTED RICE**	Grains, white roots and tubers, and plantains	3.650	0.071	0.280	0.008	0.250	1.150	0.011	0.000	0.001	0.000	0.016	0.002	0.080	0.000	0.000	0.001	0.002	0.151

Table A4 (continued)

Foods	Food Groups	Energy	Protein	Calcium	Iron	Magnesium	Phosphorus	Zinc	Vitamin C	Thiamin	Riboflavin	Niacin	Vitamin B6	Folate	Vitamin B12	Vitamin A	Vitamin E	Copper	Selenium
**LARGE ONIONS**	Other vegetables	0.400	0.011	0.230	0.002	0.100	0.290	0.002	0.074	0.000	0.000	0.001	0.001	0.190	0.000	0.000	0.000	0.000	0.005
**LOCAL RICE**	Grains, white roots and tubers, and plantains	3.580	0.065	0.030	0.008	0.230	0.950	0.011	0.000	0.001	0.000	0.016	0.002	0.060	0.000	0.000	0.000	0.002	0.000
**MAIZE**	Grains, white roots and tubers, and plantains	3.650	0.094	0.070	0.027	1.270	2.100	0.022	0.000	0.004	0.002	0.036	0.006	0.190	0.000	0.110	0.005	0.003	0.155
**MANGOES**	Vitamin A-rich vegetables and fruits	0.000	0.000	0.000	0.000	0.000	0.000	0.000	0.000	0.000	0.000	0.000	0.000	0.000	0.000	0.000	0.000	0.000	0.000
**MILLET**	Grains, white roots and tubers, and plantains	3.780	0.110	0.080	0.030	1.140	2.850	0.017	0.000	0.004	0.003	0.047	0.004	0.850	0.000	0.000	0.001	0.008	0.027
**ORANGES**	Other fruits	0.460	0.007	0.430	0.001	0.100	0.120	0.001	0.450	0.001	0.000	0.004	0.001	0.170	0.000	0.110	0.002	0.000	0.005
**PADDY RICE**	Grains, white roots and tubers, and plantains	0.000	0.000	0.000	0.000	0.000	0.000	0.000	0.000	0.000	0.000	0.000	0.000	0.000	0.000	0.000	0.000	0.000	0.000
**PALM OIL**	Other oils and fats	8.840	0.000	0.000	0.000	0.000	0.000	0.000	0.000	0.000	0.000	0.000	0.000	0.000	0.000	0.000	0.159	0.000	0.000
**PINEAPPLE**	Other fruits	0.500	0.005	0.130	0.003	0.120	0.080	0.001	0.478	0.001	0.000	0.005	0.001	0.180	0.000	0.030	0.000	0.001	0.001
**PLANTAIN**	Grains, white roots and tubers, and plantains	1.220	0.013	0.030	0.006	0.370	0.340	0.001	0.184	0.001	0.001	0.007	0.003	0.220	0.000	0.560	0.001	0.001	0.015

Table A4 (continued)

Foods	Food Groups	Energy	Protein	Calcium	Iron	Magnesium	Phosphorus	Zinc	Vitamin C	Thiamin	Riboflavin	Niacin	Vitamin B6	Folate	Vitamin B12	Vitamin A	Vitamin E	Copper	Selenium
**SALTED DRIED TILAPIA FISH**	Meat, poultry and fish	0.960	0.201	0.100	0.006	0.270	1.700	0.003	0.000	0.000	0.001	0.039	0.002	0.240	0.016	0.000	0.004	0.001	0.418
**SMOKED HERRINGS**	Meat, poultry and fish	1.580	0.180	0.570	0.011	0.320	2.360	0.010	0.007	0.001	0.002	0.032	0.003	0.100	0.137	0.280	0.011	0.001	0.365
**SORGHUM**	Grains, white roots and tubers, and plantains	3.290	0.106	0.130	0.034	1.650	2.890	0.017	0.000	0.003	0.001	0.037	0.004	0.200	0.000	0.000	0.005	0.003	0.122
**SOYBEANS**	Pulses	0.000	0.000	0.000	0.000	0.000	0.000	0.000	0.000	0.000	0.000	0.000	0.000	0.000	0.000	0.000	0.000	0.000	0.000
**TOMATOES**	Other vegetables	0.180	0.009	0.100	0.003	0.110	0.240	0.002	0.137	0.000	0.000	0.006	0.001	0.150	0.000	0.420	0.005	0.001	0.000
**YAM**	Grains, white roots and tubers, and plantains	1.180	0.015	0.170	0.005	0.210	0.550	0.002	0.171	0.001	0.000	0.006	0.003	0.230	0.000	0.070	0.004	0.002	0.007

Note: Food groups apply the definition of MDD-W. Food composition data sources include National Nutrient Database for Standard Reference (USDA) and FAO's West African Food Composition Table. Data shown are nutrients contents per gram of food items. Unit is kcal for dietary energy, g for protein, mg for calcium, iron, magnesium, phosphorus, zinc, vitamin C, thiamin, riboflavin, niacin, vitamin B6, folate, and vitamin E; and mcg for vitamin B12, vitamin A, copper and selenium.

Table A5. Food Items, Food Groups and Food Compositions (Tanzania)

Foods	Food Groups	Energy	Protein	Calcium	Iron	Magnesium	Phosphorus	Zinc	Vitamin C	Thiamin	Riboflavin	Niacin	Vitamin B6	Folate	Vitamin B12	Vitamin A	Vitamin E	Copper	Selenium
**APPLES (IMPORTED)**	Other fruits	0.520	0.003	0.060	0.001	0.050	0.110	0.000	0.046	0.000	0.000	0.001	0.000	0.030	0.000	0.030	0.002	0.000	0.000
**AVOCADO**	Other fruits	1.600	0.020	0.120	0.006	0.290	0.520	0.006	0.100	0.001	0.001	0.017	0.003	0.810	0.000	0.070	0.021	0.002	0.004
**SOYBEANS (BLANKETI)**	Pulses	4.460	0.365	2.770	0.157	2.800	7.040	0.049	0.060	0.009	0.009	0.016	0.004	3.750	0.000	0.010	0.009	0.017	0.178
**BEEF SAUSAGE**	Meat, poultry and fish	4.050	0.155	0.150	0.015	0.130	1.850	0.029	0.007	0.000	0.001	0.032	0.002	0.050	0.020	0.250	0.005	0.001	0.000
**BEEF WITHOUT BONES (STEAK)**	Meat, poultry and fish	2.780	0.175	0.080	0.019	0.170	1.560	0.036	0.000	0.001	0.002	0.035	0.003	0.070	0.027	0.000	0.000	0.001	0.159
**BITTER TOMATOES (NYANYA CHUNGU)**	Other vegetables	0.250	0.010	0.090	0.002	0.140	0.240	0.002	0.022	0.000	0.000	0.006	0.001	0.220	0.000	0.010	0.003	0.001	0.003
**BRINJALS/EGG PLANT (BILINGANYA)**	Other vegetables	0.250	0.010	0.090	0.002	0.140	0.240	0.002	0.022	0.000	0.000	0.006	0.001	0.220	0.000	0.010	0.003	0.001	0.003
**CABBAGES**	Other vegetables	0.410	0.009	0.330	0.003	0.120	0.350	0.002	0.059	0.001	0.001	0.010	0.001	0.190	0.000	8.350	0.007	0.000	0.001
**CARROTS**	Vitamin A-rich vegetables and fruits	0.410	0.009	0.330	0.003	0.120	0.350	0.002	0.059	0.001	0.001	0.010	0.001	0.190	0.000	8.350	0.007	0.000	0.001
**CASSAVA FLOUR**	Grains, white roots and tubers, and plantains	3.350	0.019	1.380	0.015	0.450	1.020	0.007	0.040	0.001	0.001	0.012	0.002	0.470	0.000	0.000	0.004	0.002	0.000
**CASSAVA FRESH**	Grains, white roots and tubers, and plantains	1.600	0.014	0.160	0.003	0.210	0.270	0.003	0.206	0.001	0.000	0.009	0.001	0.270	0.000	0.010	0.002	0.001	0.007
**COCONUT MATURE (ISIYOVUNJWA)**	Other fruits	1.620	0.016	0.190	0.022	0.170	1.110	0.004	0.020	0.001	0.001	0.006	0.000	0.120	0.000	0.000	0.003	0.002	0.000

Table A5 (continued)

Foods	Food Groups	Energy	Protein	Calcium	Iron	Magnesium	Phosphorus	Zinc	Vitamin C	Thiamin	Riboflavin	Niacin	Vitamin B6	Folate	Vitamin B12	Vitamin A	Vitamin E	Copper	Selenium
**COOKING BANANA GREEN**	Grains, white roots and tubers, and plantains	1.220	0.013	0.030	0.006	0.370	0.340	0.001	0.184	0.001	0.001	0.007	0.003	0.220	0.000	0.560	0.001	0.001	0.015
**COOKING FAT**	Other oils and fats	7.170	0.009	0.240	0.000	0.020	0.240	0.001	0.000	0.000	0.000	0.000	0.000	0.030	0.002	6.840	0.023	0.000	0.010
**COOKING OIL VARIETY**	Other oils and fats	8.840	0.000	0.000	0.000	0.000	0.000	0.000	0.000	0.000	0.000	0.000	0.000	0.000	0.000	0.000	0.382	0.000	0.000
**DRIED SARDINES (DAGAA)**	Meat, poultry and fish	1.500	0.280	1.030	0.023	0.460	3.670	0.023	0.000	0.000	0.002	0.109	0.013	0.050	0.143	0.250	0.011	0.001	0.000
**FINGER MILLET GRAINS (PUNJE ZA ULEZI)**	Grains, white roots and tubers, and plantains	3.780	0.110	0.080	0.030	1.140	2.850	0.017	0.000	0.004	0.003	0.047	0.004	0.850	0.000	0.000	0.001	0.008	0.027
**FRESH COW MILK**	Dairy	0.610	0.032	1.130	0.000	0.100	0.840	0.004	0.000	0.000	0.002	0.001	0.000	0.050	0.005	0.460	0.001	0.000	0.037
**GOAT MEAT**	Meat, poultry and fish	1.090	0.206	0.130	0.028	0.000	1.800	0.040	0.000	0.001	0.005	0.038	0.000	0.050	0.011	0.000	0.000	0.003	0.088
**GREEN PEAS (NJEGERE - ZILIZOMENYWA)**	Pulses	0.420	0.028	0.430	0.021	0.240	0.530	0.003	0.600	0.002	0.001	0.006	0.002	0.420	0.000	0.540	0.004	0.001	0.007
**GREEN PEPPER (HOHO)**	Other vegetables	0.200	0.009	0.100	0.003	0.100	0.200	0.001	0.804	0.001	0.000	0.005	0.002	0.100	0.000	0.180	0.004	0.001	0.000
**INDUSTRIALLY BRED LIVE CHICKEN - (BROILERS)**	Meat, poultry and fish	2.130	0.183	0.110	0.013	0.200	1.490	0.015	0.026	0.001	0.002	0.066	0.003	0.300	0.011	2.320	0.000	0.001	0.118
**LADIES FINGER/OKRA (BAMIA)**	Other vegetables	0.330	0.019	0.820	0.006	0.570	0.610	0.006	0.230	0.002	0.001	0.010	0.002	0.600	0.000	0.360	0.003	0.001	0.007
**LEMONS**	Other fruits	0.290	0.011	0.260	0.006	0.080	0.160	0.001	0.530	0.000	0.000	0.001	0.001	0.110	0.000	0.010	0.002	0.000	0.004

Table A5 (continued)

Foods	Food Groups	Energy	Protein	Calcium	Iron	Magnesium	Phosphorus	Zinc	Vitamin C	Thiamin	Riboflavin	Niacin	Vitamin B6	Folate	Vitamin B12	Vitamin A	Vitamin E	Copper	Selenium
**LENTILS (CHOROKO)**	Pulses	2.970	0.254	0.610	0.070	1.030	3.910	0.039	0.000	0.006	0.002	0.023	0.007	2.950	0.000	0.030	0.005	0.007	0.000
**LIMES**	Other fruits	0.300	0.007	0.330	0.006	0.060	0.180	0.001	0.291	0.000	0.000	0.002	0.000	0.080	0.000	0.020	0.002	0.001	0.004
**MAIZE FLOUR (WHITE)**	Grains, white roots and tubers, and plantains	3.610	0.069	0.070	0.024	0.930	2.720	0.017	0.000	0.002	0.001	0.019	0.004	0.250	0.000	0.000	0.004	0.002	0.154
**MANGOES**	Vitamin A-rich fruits and vegetables	0.600	0.008	0.110	0.002	0.100	0.140	0.001	0.364	0.000	0.000	0.007	0.001	0.430	0.000	0.540	0.009	0.001	0.006
**MARGARINE**	Other oils and fats	7.190	0.009	0.300	0.000	0.030	0.230	0.000	0.002	0.000	0.000	0.000	0.000	0.010	0.001	8.190	0.031	0.000	0.000
**MCHICHA (SPINACH)**	Dark green leafy vegetables	0.230	0.029	0.990	0.027	0.790	0.490	0.005	0.281	0.001	0.002	0.007	0.002	1.940	0.000	4.690	0.020	0.001	0.010
**NATURAL GROUNDNUTS (UN ROASTED)**	Nuts and seeds	5.670	0.258	0.920	0.046	1.680	3.760	0.033	0.000	0.006	0.001	0.121	0.003	2.400	0.000	0.000	0.083	0.011	0.072
**ONE EGG LAYERS**	Eggs	1.430	0.126	0.560	0.018	0.120	1.980	0.013	0.000	0.000	0.005	0.001	0.002	0.470	0.009	1.600	0.011	0.001	0.307
**ONE EGG TRADITIONAL**	Eggs	1.430	0.126	0.560	0.018	0.120	1.980	0.013	0.000	0.000	0.005	0.001	0.002	0.470	0.009	1.600	0.011	0.001	0.307
**ORANGES**	Other fruits	0.460	0.007	0.430	0.001	0.100	0.120	0.001	0.450	0.001	0.000	0.004	0.001	0.170	0.000	0.110	0.002	0.000	0.005
**PAPAYA – PAWPAW**	Vitamin A-rich fruits and vegetables	0.430	0.005	0.200	0.003	0.210	0.100	0.001	0.609	0.000	0.000	0.004	0.000	0.370	0.000	0.470	0.003	0.000	0.006
**PINEAPPLES**	Other fruits	0.500	0.005	0.130	0.003	0.120	0.080	0.001	0.478	0.001	0.000	0.005	0.001	0.180	0.000	0.030	0.000	0.001	0.001
**PORK MEAT**	Meat, poultry and fish	3.760	0.139	0.190	0.007	0.130	1.550	0.016	0.004	0.006	0.002	0.038	0.003	0.040	0.006	0.020	0.003	0.001	0.284

Table A5 (continued)

Foods	Food Groups	Energy	Protein	Calcium	Iron	Magnesium	Phosphorus	Zinc	Vit C	Thiamin	Riboflavin	Niacin	Vit B6	Folate	Vit B12	Vit A	Vit E	Copper	Selenium
**POTATOES - ROUND/IRISH**	Grains, white roots and tubers, and plantains	0.770	0.021	0.120	0.008	0.230	0.570	0.003	0.197	0.001	0.000	0.011	0.003	0.150	0.000	0.000	0.000	0.001	0.004
**POWDERED MILK**	Dairy	4.950	0.259	9.680	0.007	0.910	7.350	0.035	0.113	0.003	0.013	0.007	0.003	0.390	0.033	2.280	0.005	0.001	0.000
**RED DRY BEANS**	Pulses	3.370	0.225	0.830	0.067	1.380	4.060	0.028	0.045	0.006	0.002	0.021	0.004	3.940	0.000	0.000	0.002	0.007	0.032
**RICE**	Grains, white roots and tubers, and plantains	3.600	0.066	0.090	0.008	0.350	1.080	0.012	0.000	0.001	0.000	0.016	0.001	0.090	0.000	0.000	0.000	0.001	0.000
**ROUND ONIONS**	Other vegetables	0.400	0.011	0.230	0.002	0.100	0.290	0.002	0.074	0.000	0.000	0.001	0.001	0.190	0.000	0.000	0.000	0.000	0.005
**SWEET POTATOES**	Grains, white roots and tubers, and plantains	0.860	0.016	0.300	0.006	0.250	0.470	0.003	0.024	0.001	0.001	0.006	0.002	0.110	0.000	7.090	0.003	0.002	0.006
**SWEET BANANA**	Other fruits	0.890	0.011	0.050	0.003	0.270	0.220	0.002	0.087	0.000	0.001	0.007	0.004	0.200	0.000	0.030	0.001	0.001	0.010
**TOMATOES RED**	Other vegetables	0.180	0.009	0.100	0.003	0.110	0.240	0.002	0.137	0.000	0.000	0.006	0.001	0.150	0.000	0.420	0.005	0.001	0.000
**TRADITIONALLY BRED LIVE CHICKEN (KUKU WA KIENYEJI)**	Meat, poultry and fish	2.130	0.183	0.110	0.013	0.200	1.490	0.015	0.026	0.001	0.002	0.066	0.003	0.300	0.011	2.320	0.000	0.001	0.118
**WHEAT FLOUR**	Grains, white roots and tubers, and plantains	3.640	0.103	0.150	0.012	0.220	1.080	0.007	0.000	0.001	0.000	0.013	0.000	0.260	0.000	0.000	0.001	0.001	0.339
**WHITE MAIZE GRAINS**	Grains, white roots and tubers, and plantains	3.650	0.094	0.070	0.027	1.270	2.100	0.022	0.000	0.004	0.002	0.036	0.006	0.000	0.000	0.000	0.000	0.003	0.155

Note: Food groups apply the definition of MDD-W. Food composition data sources include National Nutrient Database for Standard Reference (USDA) and FAO's West African Food Composition Table. Data shown are nutrients contents per gram of food items. Unit is kcal for dietary energy, g for protein, mg for calcium, iron, magnesium, phosphorus, zinc, vitamin C, thiamin, riboflavin, niacin, vitamin B6, folate, and vitamin E; and mcg for vitamin B12, vitamin A, copper and selenium.
